# De novo *TRPV4* Leu619Pro variant causes a new channelopathy characterised by giant cell lesions of the jaws and skull, skeletal abnormalities and polyneuropathy

**DOI:** 10.1136/jmedgenet-2020-107427

**Published:** 2021-03-08

**Authors:** Aviel Ragamin, Carolina C Gomes, Karen Bindels-de Heus, Renata Sandoval, Angelia V Bassenden, Luciano Dib, Fernando Kok, Julieta Alves, Irene Mathijssen, Evita Medici-Van den Herik, Robert Eveleigh, Tenzin Gayden, Bas Pullens, Albert Berghuis, Marjon van Slegtenhorst, Martina Wilke, Nada Jabado, Grazia Maria Simonetta Mancini, Ricardo Santiago Gomez

**Affiliations:** 1 Department of Clinical Genetics, Erasmus MC University Medical Center, Rotterdam, The Netherlands; 2 Department of Pathology, Biological Sciences Institute, Universidade Federal de Minas Gerais (UFMG), Belo Horizonte, Brazil; 3 Department of Pediatrics, Erasmus MC University Medical Center, Rotterdam, The Netherlands; 4 ENCORE Expertise Center for Neurodevelopmental Disorders, Erasmus MC University Medical Center, Rotterdam, The Netherlands; 5 Oncogenetics, Hospital Sírio-Libanês, Brasília, Hospital Sirio-Libanes, Sao Paulo, Brazil; 6 Department of Biochemistry, McGill University, Montreal, Quebec, Canada; 7 Post Graduation Program, School of Dentistry, Paulista University (UNIP), Sao Paulo, Brazil; 8 Department of Neurology, Universidade de Sao Paulo, Sao Paulo, Brazil; 9 Division of Neurosurgery, Universidade Federal de São Paulo, São Paulo, Brazil; 10 Department of Plastic and Reconstructive Surgery, Erasmus MC University Medical Center, Rotterdam, The Netherlands; 11 Department of Child Neurology, Erasmus MC University Medical Center, Rotterdam, The Netherlands; 12 Canadian Centre for Computational Genomics (C3G), Montreal, Québec, Canada; 13 McGill University and Genome Quebec Innovation Centre, Montreal, Quebec, Canada; 14 Department of Human Genetics, McGill University Faculty of Medicine, Montreal, Québec, Canada; 15 Department of Otorhinolaryngology and Head and Neck Surgery, Erasmus MC University Medical Center, Rotterdam, Zuid-Holland, The Netherlands; 16 Department of Pediatrics, McGill University and McGill University Heath Centre Research Institute, Montreal, Quebec, Canada; 17 Department of Oral Surgery and Pathology, Universidade Federal de Minas Gerais, Belo Horizonte, Brazil

**Keywords:** genetics, medical, nervous system diseases, surgery, plastic, stomatognathic diseases, pathology

## Abstract

**Background:**

Pathogenic germline variants in *T*ransient *R*eceptor *P*otential *V*anilloid 4 *C*ation *C*hannel (*TRPV4*) lead to channelopathies, which are phenotypically diverse and heterogeneous disorders grossly divided in neuromuscular disorders and skeletal dysplasia. We recently reported in sporadic giant cell lesions of the jaws (GCLJs) novel, somatic, heterozygous, gain-of-function mutations in *TRPV4*, at Met713.

**Methods:**

Here we report two unrelated women with a de novo germline p.Leu619Pro *TRPV4* variant and an overlapping systemic disorder affecting all organs individually described in TRPV4 channelopathies.

**Results:**

From an early age, both patients had several lesions of the nervous system including progressive polyneuropathy, and multiple aggressive giant cell-rich lesions of the jaws and craniofacial/skull bones, and other skeletal lesions. One patient had a relatively milder disease phenotype possibly due to postzygotic somatic mosaicism. Indeed, the *TRPV4* p.Leu619Pro variant was present at a lower frequency (variant allele frequency (VAF)=21.6%) than expected for a heterozygous variant as seen in the other proband, and showed variable regional frequency in the GCLJ (VAF ranging from 42% to 10%). In silico structural analysis suggests that the gain-of-function p.Leu619Pro alters the ion channel activity leading to constitutive ion leakage.

**Conclusion:**

Our findings define a novel polysystemic syndrome due to germline *TRPV4* p.Leu619Pro and further extend the spectrum of *TRPV4* channelopathies. They further highlight the convergence of *TRPV4* mutations on different organ systems leading to complex phenotypes which are further mitigated by possible post-zygotic mosaicism. Treatment of this disorder is challenging, and surgical intervention of the GCLJ worsens the lesions, suggesting the future use of MEK inhibitors and TRPV4 antagonists as therapeutic modalities for unmet clinical needs.

## Introduction

The transient receptor potential vanilloid 4 cation channel (*TRPV4*) gene (OMIM #605427) codes for a Ca^2+^-permeable channel.^1^ This polymodal Ca^2+^-permeable channel is largely expressed, including in the nervous system and bone tissues, and can be activated by diverse stimuli, including heat, cell swelling, mechanical, endogenous and exogenous chemical stimuli.^2^

The discovery of causative, de novo heterozygous, gain-of-function germline *TRPV4* mutations in a wide spectrum of clinical entities leading to increased/abnormal activity of the TRPV4 channel led to the label of *TRPV4* channelopathies.^3 4^ These germline *TRPV4* channelopathies are traditionally divided based on their phenotype in neuromuscular disorders and skeletal dysplasia, with large phenotypical diversity within a given clinical entity.^4^ The neuromuscular disorders include Charcot-Marie-Tooth disease type 2C (CMT2C, OMIM #606071), scapuloperoneal spinal muscular atrophy (OMIM #181405) and congenital distal spinal muscular atrophy (OMIM #600175). Common features of these neurological disorders include progressive peripheral neuropathy, laryngeal dysfunction, respiratory dysfunction and contractures of the joints.^4^

*TRPV*4 dominant mutations also define five different types of skeletal dysplasia: at the milder end of clinical phenotype is familial digital arthropathy with brachydactyly (OMIM #606835); with more systemic features are autosomal dominant brachyolmia (OMIM #113500), spondylometaphyseal dysplasia, Kozlowski type (OMIM #184252), spondyloepimetaphyseal dysplasia, Maroteaux type (pseudo-Morquio syndrome type 2, OMIM #184095), which includes also parastremmatic dwarfism (OMIM #168400) and finally metatropic dysplasia (OMIM #156530), the latter including lethal and non-lethal (mosaic) forms.^4–6^ Last, germline gain-of-function *TRPV4* mutations have also been reported in patients with femoral head osteonecrosis.^7^

Despite this dichotomy between the nervous system and bones, TRPV4 channelopathies show a level of phenotypical overlap as initially shown by Unger *et al* 8 and confirmed by others.^9 10^ For example, vocal cord paralysis, deafness and/or contractures have been reported in both CMT2C and MD. In fact, there is a lack of genotype–phenotype correlation, while several *TRPV4* pathogenic variants have been described in association with both phenotypes even if they lead predominantly to either bone disease or neurological defects when present in an individual.^4^

We recently extended the spectrum of TRPV4 channelopathies to another clinical entity, central giant cell lesion of the jaws (GCLJ), also known as giant cell granuloma. This is a benign bone lesion that can be locally aggressive and may recur, leading to major functional impact in the jaws. Histologically, these lesions are giant cell-rich lesions, with prominent mononuclear cell proliferation and haemorrhagic background.^11 12^ We described novel recurrent somatic *TRPV4* missense mutations at Met713, a juxtamembranous residue, in a subset of GCLJ. We further showed that novel *TRPV4* p.M713V/I mutants lead to increased constitutive and stimulated channel activity, in addition to sustained activation of the MAPK/ERK pathway.^13^ Notably, we identified in additional GCLJ tumours wild-type for *TRPV4* either *KRAS* or *FGFR1* gain-of-function mutations, leading us to suggest that activated MAPK/ERK pathway promotes the genesis of sporadic and syndrome-associated giant cell rich lesions.^13 14^

We describe herein the phenotype and the long-term follow-up of two unrelated 20-year-old women with an overlapping phenotype of polyneuropathy and other neurological lesions and severe skeletal defects, including multiple bone lesions, and giant cell-rich lesions affecting the jaws and other skull/facial bones. Both patients were found to carry de novo heterozygous missense *TRPV4* variants, leading to p.Leu619Pro substitution. These two individuals were traced through Genematcher.^15^

## Methods

Informed written consent for publication of images was obtained from the subjects of the study.

### Genetic analyses

#### Subject 1

Genomic single nucleotide polymorphism microarray analysis (Affymetrix GeneChip 260K Nsp1 genotyping arrays, Genome Build 36, National Center for Biotechnology Information) showed a normal female hybridisation pattern. Whole-exome sequencing (WES) was performed on DNA from blood by the department of Clinical Genetics of the Erasmus Medical Centre in the Netherlands (certified according to European ISO regulation) on the affected individual and her parents (trio analysis), as described earlier.^16^ Genomic DNA was extracted according to standard procedures and enriched with Agilent Sureselect Clinical Research Exome Capture. Samples were run on the Illumina HiSeq platform. On average, 50 million reads per exome and a mapped fraction above 98% are obtained. Average coverage is approximately 50-fold. Reads were mapped to the genome using Burrows-Wheeler Aligner (BWA) (bio-bwa.sourceforge.net). Variant detection was performed by Genome Analysis Toolkit (www.broadinstitute.org/gatk). Analysis of variants was performed in Alissa interpret (Agilent Technologies) using the Variant Calling File followed by filtering for de novo, X-linked, recessive and variants in imprinted genes in a panel of 2753 genes believed to be involved in multiple congenital anomalies. Variants were filtered on quality (read depth≥10), minor allele frequency (≥1% in 200 alleles in the Single Nucleotide Polymorphism Database (dbSNP), ESP6500, the 1000 Genome project, or the ExAC database), location (within an exon or first/last 10 bp of introns). At last, GnomAD V.3.0 was also consulted for variant interpretation. Variant classification followed the 2015 standards and guidelines of the American College of Medical Genetics and Genomics. Only variants reaching the threshold of pathogenic or likely pathogenic were reported, in this case the only reported variant concerned *TRPV*4.

#### Subject 2

WES was performed on germline blood DNA of the proband, both parents and tumour DNA from mandible and maxilla of the patient. The library was generated using the Agilent The SureSelectXT Low Input Reagent Kits according to the manufacturer’s instructions. The enriched libraries were sequenced on Illumina HiSeq 4000 with 100 bp paired-end reads. WES data were analysed for variants using GenPipes pipeline.^17^ This pipeline follows the stepwise procedures of the BROAD Institute GATK best practices. Raw reads derived from the sequencing instrument are quality trimmed and adapter clipped using Trimmomatic^18^ to obtain a high-quality set of reads for sequence alignment (sam/bam) file generation. The trimmed reads are aligned to a reference genome (build 37) using a fast, memory-efficient Burrows-Wheeler transform aligner BWA-mem.^19^ Mapped reads are further refined using Genome Analysis Toolkit (GATK)^20^ and Picard program suites (http://broadinstitute.github.io/picard) to improve mapping near insertions and deletions (indels; GATK indel realigner), remove duplicate reads with the same paired start site (Picard mark duplicates) and improve quality scores (GATK base recalibration). Variants are called using GATK haplotype caller in gvcf mode to allow efficient downstream merging of multiple samples into one variant file to streamline downstream variant processing procedures which include normalisation and decomposition of multinucleotide polymorphisms,^21^ functional annotation with SNPeff^22^ and variant annotations using the Gemini^23^ framework, which provides quality metric and extensive metadata to help further prioritise variants. Annotated variants were filtered against the common germline polymorphisms present in dbSNP135, the 1000 Genomes Project^24^ and the gnomAD database. Somatic mutations were called by comparing variants from the matched tumor-normal pairs

## Results

### Subject 1

The first subject is a 20-year-old Dutch woman born of healthy non-consanguineous parents, with negative family history. She was born at 41 weeks of gestation with normal birth weight, length and head circumference. An overview of her clinical symptoms and features is shown in table 1.

**Table 1:** Overview of clinical symptoms and features of both subjects

Symptoms dimension	Subject 1	Subject 2
Facial and skull dysmorphism	Relative macrocephaly Dolichocephaly Wide collapsed nasal bridge Hypertelorism Retrognathia Low implanted ears Short webbed neck	Ocular hypertelorism Low implanted ears Left cochlear malformation
Neurological	Vocal cord paresis Fasciculations of the tongue Atrophy of tongue and hand muscles Thoracic meningomyelocele with syrinx Tethered cord Peripheral polyneuropathy/spinal atrophy Bilateral sensineuronal hearing loss Impairment of respiratory muscles Bilateral pes cavus Normal cognitive development	Vocal cord paresis Left facial palsy Peripheral axonal polyneuropathy Progressive bilateral mixed hearing loss Bilateral pes cavus Normal cognitive development
Skeletal	Arthrogryposis multiplex congenita: adducted thumbs, camptodactyly of the second to fourth digits of the right hand, clubfeet Cystic lesions of long bones and vertebrae Giant cell lesions of the jaw and skull Thoracic vertebrae fusion Left convex thoracic scoliosis Cubitus valgus Progressive contractures of large joints Short stature (<−5 SD)	Bone lesions (femur and foot) Giant cell lesions of the jaws and skull Cervical bone fusion Scoliosis Limb asymmetry
Pulmonary	Recurrent respiratory infections Asthma Impaired lung function	None
Urological	Vesicoureteral reflux and hydronephrosis (neurogenic bladder)	None
Ocular	Chronic blepharitis Hyperopia Papilledema	Pigmentary retinopathy
Other	None	Asymptomatic hyperplasia of the parathyroid glands Bilateral inguinal hernia

At birth, dysmorphic features were noted. She had a wide collapsed nasal bridge, retrognathia, low implanted ears, a short webbed neck, a prominent occiput, a high pitched cry, bilateral congenital clubfeet, distal clasped thumbs, camptodactyly of the second to fourth digits of the right hand, absence of the bending folds of the distal interphalangeal joints, joint contractures of the left digits and a vascular lesion on the forehead. Genetic screening revealed a normal female karyotype.

Soon after birth, a skeletal survey showed no abnormalities except for possible mild vertebral anomalies at the cervical and upper thoracic level. At the same time, airways obstruction was noted, which later appeared to be caused by vocal cord paresis and required a tracheostomy at 8 months. Feeding difficulties were also present and required gastrostomy tube feeding at the age of 2.5 years.

**Figure 1:**
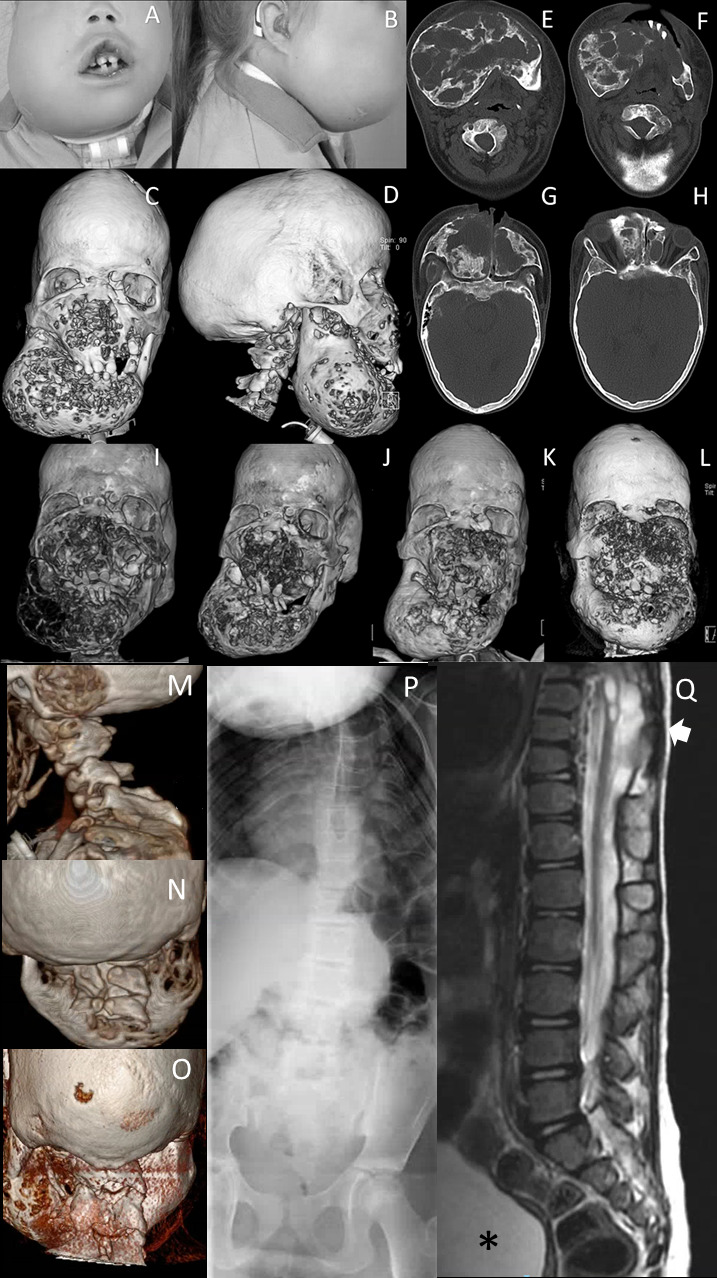
Clinical and radiological facial features of subject 1. (A, B) Facial photographs at age 11. Dysmorphic features include frontal bossing, dolichocephaly, hypertelorism, half-closed eyes, a broad and flat nasal bridge, and an asymmetric bilateral swelling at mandibular and mid-facial levels. (C, D) 3-D reconstructed CT images of the face at age 11 years show a bilateral asymmetric cystic expansion of the mandible, maxilla, ethmoid and frontal bones with medial displacement of the teeth at mandibular level. In addition, frontal bossing and dolichocephaly can be noted. (E–H) Transverse CT images of at mandibular and mid-facial levels at age 11 years show extensive osteolytic and osteoblastic lesions. Note that the lesions are not only at mandibular level but also on mid-facial level and the anterior side of the foramen magnum. (I) 3-D reconstructed CT image of the face at age 9 years shows a bilateral asymmetric cystic expansion of the mandible, maxilla, ethmoid and frontal bones with medial displacement of the teeth and bilateral orbital involvement. (J) One year after the start of pamidronate (age 11 years), small cystic lesions can be seen at mandibular and mid-facial level; note the difference in osseous tissue compared with the CT scan before the start of the therapy. (K) Five years after the start of therapy (age 14 years), more remodelling of osseous tissue has occurred, although small cystic lesions are present. (L) One year after stopping pamidronate and 2 years after shaving approximately 2.5 cm of the right maxilla (age 17 years), multiple cystic lesions at mandibular and mid-facial levels with intensive displacement of the orbita. CT 3-D reconstruction of the skull, with (M) a sagittal view and (N) a posterior view of the cervical spine showing, besides lytic lesions, abnormalities of the cervical vertebrae at the age of 6 years and (O) at 18 years. (P) Spine X-rays at the age of 7 years showing scoliosis and abnormal thoracic vertebrae. (Q) Sagittal T2-weighted MRI of the thoracolumbal spine showing the thoracic syrinx and meningomyelocele (arrow) and neurogenic bladder (asterisk).

From the first year, she had recurrent pyelonephritis. Further screening showed hydronephrosis and vesicoureteral reflux leading to progressive renal failure. The cause was found in a detrusor sphincter dyssynergia. Spine MRI showed thoracic subcutaneous meningomyelocele and a syrinx at the 9th and 10th vertebrae, a tethered cord with a medullary cone at the fourth lumbar vertebra, leading to the diagnosis of neurogenic bladder (figure 1). After surgery, the patient regained sensibility in the saddle area but remained lifelong incontinent.

At the age of 2 years, progressive outgrowth of fibrous tissue at mandibular and mid-facial level was noted. The CT scan showed multiple asymmetric cystic lesions at mandibular and mid-face levels. Due to functional complaints (papilledema and occlusion of the larynx), surgical reduction of the fibrous tissue was performed on several occasions. This gave transient improvement, but regrowth occurred, requiring repeat surgeries for symptom relief (figure 1A–L). Histological examination was most suspect for central giant cell granuloma. Her growth lagged behind and short stature was reported at the age of 3 years, when the head circumference was 53.5 cm (+2.56 SD).

She was examined by the neurologist at the age of 6 years: except for a high-pitched voice, cubitus valgus, and fixed contractures of fingers and toes, neurological examination at that time did not show signs of a neuromuscular disorder. Her head circumference was 54.7 cm (+2.21 SD). Her psychomotor development was normal. Brain MRI showed enlargement of pericerebral cerebrospinal fluid spaces, which resolved at later imaging, and normal brain structures and ventricles. A flexible laryngoscopy confirmed bilateral vocal cord paresis with impaired abduction. Brainstem evoked response audiometry revealed deafness on the left side and a sensorineural hearing loss of 50 dB on the right side for which she received a hearing aid.

Total body skeletal survey at the ages of 7 and 9 years found cystic lucencies on both clavicles and distal humerus, fusion of the first and second thoracic vertebrae, interruption of the posterior elements of the vertebrae at cervical and upper thoracic levels, a ‘trophic’ disorder of the distal femur, and levoconvex scoliosis and proportioned short stature (figure 1M–Q). On later revision, the skeletal lesions were not sufficient to be classified as one of the TRPV4-related dysplasias, although some features resembled mild brachyolmia.

In addition to surgical debulking of skull lesions, treatment with calcitonin, then pamidronate based on calcitonin side effects, were initiated and gave pain relief while stabilising the osteolytic processes.

**Figure 2:**
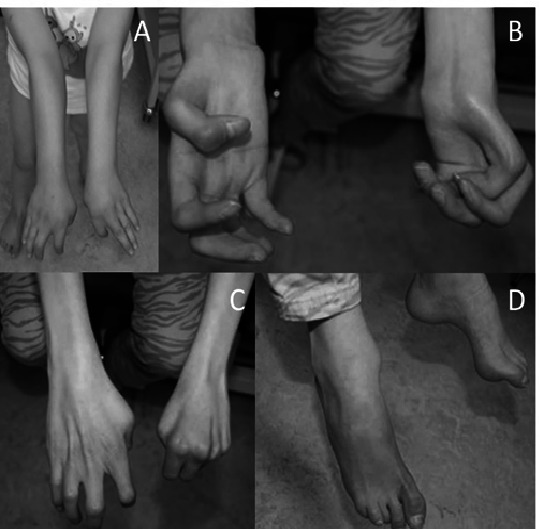
Clinical features of subject 1 at the age of 6 years (A) and 16 years (B–D), showing cubitus valgus, progressive contractures of metacarpophalangeal and interphalangeal joints, contractures of the toes and pes cavus.

At age 15 years, she required ventilation at night because of unexplained hypoventilation/central apnoea. Repeat neurological examination at this age revealed impaired sensibility, minimal gnostic impairment and low reflexes of the lower extremities, interpreted as signs of polyneuropathy, and fasciculation and atrophy of the tongue, atrophy and loss of strength in the hands, ascribed to motor neuron disease; the central sleep apnoea and the neurogenic bladder were ascribed to central nervous system dysfunction. Ophthalmological examination showed high hypermetropia, corneal astigmatism and normal fundus. At the last physical examination at age 20 years, she has a short stature (138 cm, below −5 SD), low weight (35 kg, below −4.33 SD) and a normal occipitofrontal circumference (56.2 cm, +0.78 SD), short barrel-shaped trunk, severe levoconvex scoliosis, cubitus valgus with limited extension, fixed contractures of hands and feet phalanges, mild contracture of the knees, bilateral pes cavus and areflexia of the limbs (figure 2). The skull CT revealed additional lytic lesions in the frontal, temporal and sphenoidal bones. Communication was limited by the tracheal cannula and hearing loss. Puberty and sexual development were normal. She graduated from high school and is a second year student at a university college. At the time of publication, she lives at home and receives intensive home care.

Several laboratory investigations were performed. Since fibrous osseous dysplasia has been described in cherubism and sporadically in Noonan syndrome, sequencing of exon 9 of the *SH3BP2* or *KRAS*, *BRAF* and *PTPN11* genes was performed, but no pathogenic variant was detected. Genomic microarrays showed a normal female pattern. Trio full-exome sequencing in DNA from blood detected a de novo variant c.1856_1857delinsCT (p.(Leu619Pro) in the *TRPV4* gene (NM_021625.4). The total number of reads for this codon was 26, with seven wild-type reads and 19 times delinsCT (VAF=73%). The data were interpreted as constitutional (germline) heterozygosity for the *TRPV4* variant. No other variants of unknown significance were found.

### Subject 2

The second subject is a 20-year-old Brazilian woman born to healthy non-consanguineous parents. She was born by caesarean delivery after a full-term pregnancy with normal weight, length and head circumference. An overview of her clinical symptoms and features is shown in table 1. Gastro-oesophageal reflux was described in the neonatal period. She had no significant dysmorphic features at birth. Episodes of transient respiratory discomfort leading to dyspnoea during laughing and crying occurred in early childhood. At the age of 3 years, she was admitted to the intensive care unit for respiratory distress following a laryngoscopy performed for laryngeal stridor evaluation. This procedure revealed a paradoxical movement of the vocal cords attributed to vocal cord paralysis. Otorhinolaryngology investigation, including mastoid tomography, also identified hypoplasia of the left cochlea, the left cochlear modiolus and the left cochlear round window. Periodic audiometry and brainstem auditory evoked potential showed mixed hearing loss in the right ear (auditory threshold 250–2000 Hz) and progressive sensorineural hearing loss in the left ear. Other findings included left facial palsy and hoarse voice. Some ophthalmological findings were described such as hyperopia, astigmatism, and left eye amblyopia. Funduscopy revealed bilateral retinal dystrophy with diffuse atrophy of the retinal pigment epithelium and blurring of the papillae.

**Figure 3:**
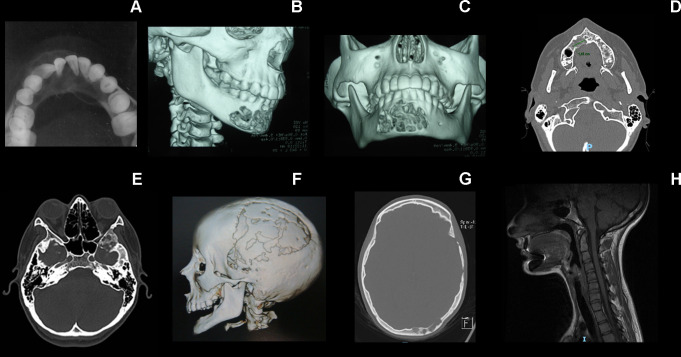
Radiological features of individual 2’s skeletal alterations. (A) Multilocular radiolucent tumour in the anterior part of the mandible causing teeth displacement. (B, C) CT image with 3-D reconstruction showing cortical bone destruction. (D) Axial CT scan showing the primary tumour in the maxilla. (E–G) Osteolytic lesions in the squamous part of the temporal bone, greater wing of the sphenoid, lateral wall of the orbit and diploe of the left occipital bone. (H) Hypoplasia of the vertebral bodies and the intervertebral disc of C2–C3, with fusion of its posterior elements characterising vertebra in block.

**Figure 4:**
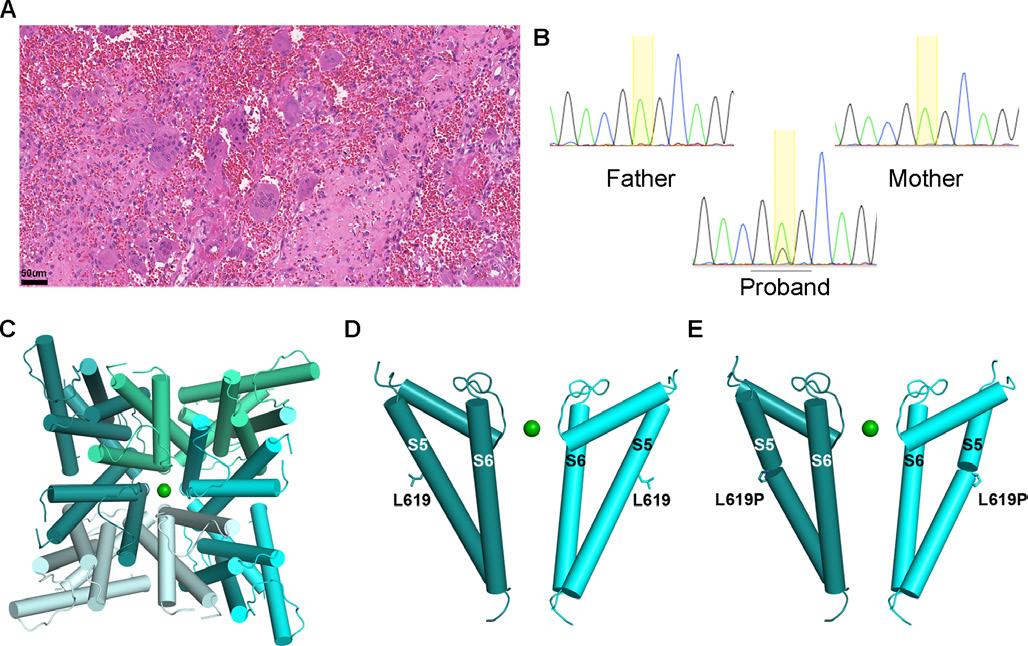
Photomicrograph of a mandibular giant cell lesion and screenshots of chromatograms of individual 2. (A) Subject 2: histopathological features of the mandibular tumour displaying numerous giant cells in a fibroblastic and haemorrhagic stroma (standard H&E staining, magnification bar: 50 μm). (B) Subject 2: screenshots of Sanger sequencing chromatograms showing the *TRPV4* c.1856T>C (p.Leu619Pro) in blood DNA, which was detected in the proband and was absent in both parents. The proportion of the variant allele and wild-type allele peaks is consistent with a variant allele frequency of 21.6% detected in the whole-exome sequencing. (C) Pore view of homotetrameric TRPV4 transmembrane domain in the presence of barium (PDB ID: 6C8G) (D). Channel view of pore domain S5–S6 of TRPV4 showing two opposing subunits for clarity, with Leu619 and (E) Leu619Pro shown in stick representation. TRPV4, transient receptor potential vanilloid 4 cation channel.

A bone lesion in the mandible was first detected at the age of 7 years (figure 3A–C). Histological examination showed a giant cell lesion compatible with GCLJ diagnosis (figure 4A). Other lesions were detected in different sites during follow-up, including skull, mandible and orbit (figure 3E–G). Several surgical interventions were performed, where lesions were debulked, but all recurred. Left craniectomy for resection of the cranial lesion was performed and the skull convexity was reconstructed with a titanium mesh. Then, a left pterional craniectomy was performed with extension to the left orbit. An anatomical prosthesis was placed during the same surgical time, which showed the inner table of the skull had visibly expanded without involvement of the underlying dura. In addition to surgery, she received pamidronate for 1 year with no impact on the progression of the bone lesions. Recently, new bone lesions were detected in the right femur and cuneiform bone of the left foot. Both are under observation. Also, a maxillary lesion between the teeth 12 and 13, and recurrence of the mandibular lesions were noted, and both were resected as surgically accessible (figure 3D).

Weakness of the left lower limb and cramps were noted at the age of 4 years. Routine ophthalmological evaluation at the age of 5 years revealed pigmentary retinopathy. At the age of 12 years, physical examination disclosed convergent non-paralytic strabismus, bilateral pes cavus, distal wasting of hands and feet and stork legs. Neurological examination showed normal cognitive function. She had a gait disturbance due to steppage and a bilateral weakness, affecting the four limbs but more intense in the left lower limb. Deep tendon reflexes were universally abolished. No sensory abnormality was present.

Nerve conduction studies demonstrated motor axonal damage in the upper and lower limbs. Surgical intervention for Achilles tendon contracture of the left lower limb was necessary at the age of 15 years. During puberty, she presented with progressive scoliosis mainly due to her limb asymmetry but with no indication of surgical intervention. Other minor skeletal findings were described, such as hypoplasia of the C2–C3 vertebral bodies, with fusion of its posterior elements, including C1 (figure 1H). During childhood, her growth curve was at the lower limit of normality (SD −2), but during puberty, she reached her familiar target height, and her final height was 154 cm (SD <-1, >−2). Her mother’s height was 156 cm and the father’s height was 175 cm; the expected familiar target height for her was 159 cm (175–13+156/2) with deviation of + or −9 cm.

Hereditary polyneuropathy with early-onset axonal neuropathy with vocal cord involvement was suspected and targeted Sanger sequencing of *TRPV4* in blood DNA revealed the exon 12 variant c.1856T>C (NM_021625.5) leading to p.(Leu619Pro) in the proband, but absent in both parents (figure 4B). Leucine at codon 619 is highly conserved among species and its substitution by proline is not present in any population database (GnomAD, 1000 Genomes and Brazilian Genomic Variants (AbraOM)) or variant repositories (HGMD, ClinVar). According to American College of Medical Genetics recommendations, this variant was classified as likely pathogenic. WES was carried out on germline blood DNA from the proband and parents, as well as on a the primary and the recurrent mandibular tumour, in addition to the maxillary GCLJ. *TRPV4* c.1856T>C, leading to p.Leu619Pro, was detected in the proband blood (VAF=21.6%, 11/51 reads). The parental exomes were negative for the mutation, consistent with a de novo variant. The low VAF for this novel variant is suggestive of postzygotic somatic mosaicism, and indeed WES of two GCLJ revealed variable variant frequencies, with a higher VAF being observed in the more aggressive and recurrent mandibular tumour (VAF=42%, 24/57 reads) in comparison with the less aggressive primary maxillary lesion (VAF=10%, 6/60 reads).

The relatively milder phenotype of subject 2 when compared with subject 1 is in line with possible somatic mosaicism suggested by sequencing.

### TRPV4 variant modelling

TRPV4 is a ubiquitous transmembrane tetrameric protein with several functional domains, among which ankyrin repeat domains (ANK1-6), preceded by a proline-rich domain, and followed by six transmembrane segments (S1–S6) and a pore-forming region S5 and S6. The C-terminus contains a calmodulin-binding site.^4^ To predict whether the p.Leu619Pro mutation would affect TRPV4’s function, its positioning concerning the channel pore was examined using a previously published structure of TRPV4 from *Xenopus tropicalis*, which shares a 78% sequence identity with the human protein, where Leu619 is conserved (figure 4C).^25^ Based on this structure, residue Leu619 is located in the centre of helix S5, adjacent to the pore-lining helix S6 (figure 4D). The Leu619Pro mutation on S5 would cause this helix to exhibit a minor kink, affecting the packing interface between S5 and S6 (figure 4E). Based on this analysis, the Leu619Pro mutation is predicted to trigger S6 movement, allowing for increased ion permeability. *TRPV4* mutations often result in gain of function, leading to increased channel activity and Ca^2+^ influx.^7 13 26–28^ Notably, in an experimental model with rat TRPV4 expressed in *X. laevis* oocyte, p.Leu619Pro mutation resulted in a constitutively active channel.^29^ Additionally, *TRPV4* p.Leu619Phe has been suggested to have a gain-of-function effect.

## Discussion

Both unrelated individuals described here share a previously unrecognised disorder characterised by GCLJ and giant cell-rich lesions affecting facial/skull bones, skeletal changes and progressive polyneuropathy and carry novel de novo germline *TRPV4* variants leading to p.Leu619Pro substitution, expanding the spectrum of TRPV4 channelopathies.

GCLJs are benign tumours that mainly affect the mandible and occur predominantly in subjects younger than 30 years.^11^ GCLJ can be sporadic and share histological similarities with other giant-cell rich lesions that may affect the jaws, such as chondroblastoma, non-ossifying fibromas, giant cell tumours of bone. In fewer occasions, GCLJ-like lesions occur in diseases and syndromes, including cherubism (OMIM #118400), Noonan syndrome (OMIM #163950), neurofibromatosis type 1 (OMIM #162200), cardiofaciocutaneous syndrome (OMIM #115150), oculoectodermal syndrome (OMIM# 600268) and osteoglophonic dysplasia (OMIM #166250).^14^ GCLJ course is often benign, with approximately 20% recurrence rate after surgical excision.^30^ The GCLJ/skull in both of our subjects are instead very debilitating and the lesions recidivate and seem to become more aggressive after surgical intervention, causing life-threatening impairment. Management of the GCLJ and giant cell lesions of the skull remained a challenge in both affected individuals. The first individual was first treated with calcitonin and later with pamidronate, with an interval of 3 months (figure 1).^31^ Besides inhibiting osteoclasts, pamidronate has antiangiogenic properties,^32^ and it was able to slow the process down and provided relief against the pain in subject 1, but complaints recidivated in the weeks prior to the next administration of pamidronate. In subject 2, pamidronate did not prove to be clinically effective. In this report, the giant cell-rich lesions affecting the jaws and skull of both patients recurred several times and, unfortunately, they seem to worsen with each new surgical intervention.

TRPV4 is involved in intracellular Ca^2+^ regulation and has an important role in osteoclast differentiation and bone homeostasis,^33–35^ in addition to a role in modulating vascular function.^36^ Ca^2+^ influx can activate the MAPK pathway,^37^ which is keystone in the skeletal development and bone homeostasis.^38^ Recently, activation of the MAPK/ERK pathway has been shown in GCLJ,^13^ irrespective of *TRPV4*, *KRAS* or *FGFR1* mutational profiles. In vitro expression of *TRPV4* p.Met713Val/Ile mutants detected in GCLJ increased both constitutive and stimulated channel activity and led to sustained activation of the ERK1/2. Such *TRPV4* p.Met713Val/Ile mutant effects could be prevented by TRPV4 antagonists.^13^ *TRPV4* also has central importance in hereditary neuropathies, and the pathophysiological mechanisms involved in such processes are still poorly understood.^4^

Based on the aforementioned discussed role of TRPV4 in bone and peripheral neural tissues and the phenotypical similarities between the two reported cases, the de novo germline *TRPV4* p.Leu619Pro mutation explains the GCLJ and giant cell-rich lesions of the skull, the skeletal alterations and neurological phenotype. The WES results of the Brazilian individual were consistent with postzygotic somatic mosaicism, which is in line with the milder phenotype observed in this individual. Interestingly, somatic mosaicism for a lethal *TRPV4* mutation has been previously shown to result in non-lethal MD.^6^

Considering treatment of these patients is challenging, and surgical interventions of the GCLJ seemed to result in a worse clinical scenario, based on previous findings on GCLJ,^13^ MEK inhibitors might be clinically relevant to treat these lesions. Additionally, specific TRPV4 antagonists represent a promising treatment option for TRPV4-related diseases, some of which have been tested for several indications and are relatively safe.^39 40^ Based on the theoretical mode of action and experimental evidence pointing to minimal side effects in animal as well as in humans,^39^ we assume that this targeted treatment could be potentially useful in our subjects, reducing the need for additional surgical treatments.^41^

## Data Availability

Data are available upon reasonable request. Exome data have been generated after obtaining written consent and are stored at ISO certified diagnostic laboratories. For privacy reasons, data are not immediately publicly available.
